# Degeneracy of the First Families of Virginia

**Published:** 1854-07

**Authors:** 


Degeneracy of the First Families of Virginia.—A Rev. Charles Brookes submitted a paper to the American Statistical Association upon the effect of intermarriage between blood relations. In the course of it he stated "some of the 'first families' of Virginia have degenerated to a painful extent, on account of the repeated intermarriages of the members, in their attempts to keep the property in the family. Some of the 'best blood' has thus so degenerated, that those who now represent it are dwarfs in more than a single sense."

---

A Mr. Edmonson, in McCraken County, Ky., last week, on setting down to breakfast, discovered the biscuit on the table of an unusual color; he called his cook and required her to eat one of them, which she did very reluctantly, and died in fifteen minutes afterwards from the effects of the poison she intended for her master and mistress.

---

Clergymen in Pennsylvania.—The census of 1850, shows that in Pennsylvania there is one clergyman to every 850; one lawyer to every 924; and one physician to every 528 inhabitants.

